# A Natural Dietary Supplement with a Combination of Nutrients Prevents Neurodegeneration Induced by a High Fat Diet in Mice

**DOI:** 10.3390/nu10091130

**Published:** 2018-08-21

**Authors:** Domenico Nuzzo, Antonella Amato, Pasquale Picone, Simona Terzo, Giacoma Galizzi, Francesco Paolo Bonina, Flavia Mulè, Marta Di Carlo

**Affiliations:** 1 Istituto di Biomedicina ed Immunologia Molecolare “A. Monroy” (IBIM), Consiglio Nazionale delle Ricerche (CNR), via Ugo La Malfa 153, 90146 Palermo, Italy; domenico.nuzzo@cnr.it (D.N.); pasquale.picone@cnr.it (P.P.); giacoma.galizzi@ibim.cnr.it (G.G.); 2 Dipartimento di Scienze e Tecnologie Biologiche, Chimiche e Farmaceutiche (STEBICEF), Università di Palermo, viale delle Scienze, Edificio 16, 90128 Palermo, Italy; antonella.amato@unipa.it (A.A.); simona.terzo@hotmail.it (S.T.); flavia.mule@unipa.it (F.M.); 3 Dipartimento di Scienze del Farmaco, Università degli Studi di Catania, Viale Andrea Doria, 6, 95125 Catania, Italy; bonina@unict.it

**Keywords:** obesity, HFD mice, natural antioxidants, insulin resistance, neurodegeneration

## Abstract

Obesity and metabolic disorders can be risk factors for the onset and development of neurodegenerative diseases. The aim of the present study was to investigate the protective effects of a natural dietary supplement (NDS), containing Curcuma longa, silymarin, guggul, chlorogenic acid and inulin, on dysmetabolism and neurodegeneration in the brains of high fat diet (HFD)-fed mice. Decrease in the expression of FACL-4, CerS-1, CerS-4, cholesterol concentration and increase in the insulin receptor expression and insulin signaling activation, were found in brains of NDS-treated HFD brains in comparison with HFD untreated-mice, suggesting that NDS is able to prevent brain lipid accumulation and central insulin resistance. In the brains of NDS-treated HFD mice, the levels of RNS, ROS and lipid peroxidation, the expression of p-ERK, H-Oxy, i-NOS, HSP60, NF-kB, GFAP, IL-1β, IL-6 and CD4 positive cell infiltration were lower than in untreated HFD mice, suggesting antioxidant and anti-inflammatory effects of NDS. The decreased expression of p-ERK and GFAP in NDS-treated HFD mice was confirmed by immunofluorescence. Lastly, a lower number of apoptotic nuclei was found in cortical sections of NDS-treated HFD mice. The present data indicate that NDS exerts neuroprotective effects in HFD mice by reducing brain fat accumulation, oxidative stress and inflammation and improving brain insulin resistance.

## 1. Introduction

There has been an increase in the human life span, with the number of people over the age of 60 expected to double in the next 30 years. Accordingly, the prevalence of neurodegenerative diseases, such as Alzheimer’s disease (AD) and other forms of dementia, progressively increasing creating profound economic and social consequences.

Some evidence suggests that diet and lifestyle can play an important role in delaying the onset or progression of age-related diseases and in improving cognitive functions [1]. However, in industrialized countries, the consumption of high-fat fast food is widely diffused. A high-fat diet (HFD) has been implicated in several metabolic pathologies, such as type 2 diabetes (T2D), obesity, and non-alcoholic fatty liver disease (NAFLD), which can be risk factors for AD or other neurodegenerative diseases [2,3,4,5,6]. The content of several lipid species, such as total triglycerides, cholesterol and ceramides, has been found to be elevated in the brains of HFD-fed mice [7], and altered lipid homeostasis in the brain has been shown to be involved in the pathogenesis of neurodegenerative diseases in conditions of obesity [8,9]. 

On the other hand, brain insulin resistance has been reported to be involved in AD patients [10]. In the brain, insulin regulates glucose uptake, neuronal and glial functions, such as growth, survival, metabolism, gene expression, synapse formation and plasticity [11]. Brain insulin resistance has been associated with a reduced number of insulin receptors (IR) and impaired signaling, with biochemical and molecular consequences leading to neurodegeneration [12,13]. In addition, in HFD-fed mice, the reduced presence of IR in the brain and a defect in Akt-Foxo insulin signaling have been related to increase of plaque formation [14]. Furthermore, insulin resistance contributes to inducing several dysfunctions, such as oxidative stress, lipid peroxidation, mitochondrial dysfunction, cytokine alterations and inflammation [14]. Therefore, AD can be regarded as brain diabetes, and is sometimes referred to as “Type 3 diabetes” [15,16]. However, what triggers insulin resistance in the brain is not well understood, and peripheral factors may be involved. In pathologies such as non-alcoholic steatohepatitis (NASH), hepatic insulin resistance, oxidative stress and injury together promote the increased generation of “toxic lipids” such as ceramides [17]. The cytotoxic ceramides are transferred from the liver to the circulation, and because they can cross the blood-brain barrier (BBB), they reach the brain and thereby exert neurodegenerative effects via a liver-brain axis [18]. 

A correct lifestyle, including a healthy diet combined with regular physical exercise, could prevent both metabolic dysfunctions and related neurodegenerative diseases. In this view, the positive effects of nutraceuticals, functional foods and the Mediterranean Diet (Mediet) on health are well known [19,20,21]. A study of two Mediterranean populations, one from the island of Ikaria (Greece) with people over 90 years old, and another from the Sicani Mountains (Sicily, Italy), with a prevalence of centenarians without dementia, has attributed this longevity to the nutraceutical component of the Mediet [22,23]. Nutraceuticals, indeed, are naturally derived bioactive compounds present in foods that have medicinal properties. Natural compounds with antioxidant and anti-inflammatory properties can retard or reverse neurodegeneration, and they have been proposed as alternative therapeutic agents for neurodegenerative diseases [24,25,26,27].

Recently, a natural dietary supplement (NDS), containing extracts from *Cynara scolymus* (chlorogenic acid), *Silybum marianum* (silymarin), *Taraxacum officinale* (inulin), *Curcuma longa* (turmeric) and *Commiphora mukul* (guggul) plants, that provides hepatic protection and reduces anthropometric parameters and total cholesterol levels in patients with MetS [28] has been demonstrated to have beneficial effects against NASH and atherosclerosis in HFD obese mice through gene modulation in the liver [29]. In particular, the upregulation of genes related to anti-inflammatory activity and lipid synthesis, and the downregulation of genes linked to pro-inflammatory responses, have been demonstrated [29].

On the basis of these results, the purpose of the present study was to evaluate whether NDS can exert positive and beneficial actions in preventing neurodegeneration induced by HFD in mice. To this end, obese mice were chronically treated with NDS and simultaneously administrated a HFD for 16 weeks, and several brain lipid synthesis enzymes, central insulin resistance, markers of neuroinflammation, oxidative stress and neurodegeneration were analyzed and compared with untreated obese animals.

## 2. Materials and Methods

### 2.1. Animals

Four-week old male C57BL/6J (B6) mice, purchased from Harlan Laboratories (San Pietro al Natisone, Udine, Italy) were housed in temperature (23 ± 1 °C) and relative humidity (55 ± 5%) controlled rooms under an artificial 12 h light/dark cycle. Standard laboratory food (code 4RF25, Mucedola, Milan, Italy) and water were freely available *ad libitum*.

After one week of acclimatization, the mice were randomly divided into three groups: 1. control group (*n* = 8), fed a standard laboratory diet (STD); 2. HFD group (*n* = 8), fed a high-fat diet (code PF4051/D, Mucedola, Milan, Italy) consisting of 34% fat (providing 60% of energy), 23% protein and 38% carbohydrates (untreated-HFD); 3. HFD group (*n* = 8) that for 16 weeks received, simultaneously, the HFD and a daily administration of NDS (0.9 mg/mouse) (treated-HFD). The composition of the standard and HFD diets are shown in Table 1 and Supplemental Tables S1 and S2. The NDS dose and treatment time were chosen on the basis of a previous study showing beneficial effects against hepatic steatosis in HFD mice [29]. The commercial name of NDS is Kèpar^®^, and it was provided by Rikrea^®^ S.r.l. (Modica, Italy). The main components of NDS are plant-derived compounds (turmeric, silymarin, guggul lipids, chlorogenic acid, inulin) which are well-known for their antioxidant and anti-inflammatory properties. The ingredients of the NDS formulation have been previously reported [29].

The mice received a daily dose of freshly made NDS by oral administration, prepared as previously reported [29]. During the 16 weeks of the treatment, changes in body weight were periodically monitored and compared between the different groups of animals. At the end of treatment, all mice, after fasting overnight, were sacrificed by cervical dislocation. Blood was immediately drawn by cardiac puncture; then the entire aortic tree was perfused with Dulbecco’s phosphate-buffered saline containing 2 mM EDTA. Perfusion was carried out via a cannula introduced into the left ventricle, with incision of the right atrial appendage to permit the outflow of blood and perfused. At the end of the perfusion procedure, livers and brains were immediately explanted, weighed and processed for subsequent analysis. Animal care and handling throughout the experimental procedures were in accordance with the European Communities Council Directive of 24 November 1986 (86/609/EEC). The experimental protocols were approved by the animal welfare committee of the University of Palermo and authorized by the Ministry of Health (Rome, Italy; Authorization Number 476/2016-PR).

### 2.2. Plasma Level of Lipids and Glucose 

Basal glycemia of mice which had fasted for 6 h was measured in blood collected from the tail vein using a commercial glucometer one day before sacrifice (GlucoMen LX meter, Menarini, Italy). Lipids were analyzed in plasma obtained from blood collected by cardiac puncture, transferred into tubes containing 1 mg/mL of EDTA and centrifuged at 825 g for 10 min. Plasma triglyceride, cholesterol, low density lipoprotein (LDL), and high density lipoprotein (HDL) levels were measured using the ILAB 600 Analyzer (Instrumentation Laboratory, Bedford, MA, USA).

### 2.3. Total Protein Extraction and Western Blot

Total proteins, extracted from brain and liver tissue, were prepared by dissolving them in solubilizing buffer (50 mM Tris-HCl pH 7.4, 150 mM NaCl, 0.5% Triton X-100, 2 mM PMSF, 1 mM DTT, 0.1% SDS) with protease inhibitor (Amersham, Life Science, Les Ulis, France) and phosphatase inhibitor cocktail II (Sigma-Aldrich, Poole, Dorset, UK). Total proteins in the lysates were quantified by the Bradford method (Bio-Rad). 50 μg of protein samples were resolved by 10% SDS-PAGE and transferred onto nitrocellulose filter for Western blotting using anti-FACL4 (1:500, Novus Biologicals, Littleton, CO, USA), anti-Insulin receptor (1:500, Santa Cruz Biotechnology, Santa Cruz, CA, USA), anti-AKT (1:1000, Cell Signaling Technology, Beverly, MA, USA), anti-phospho-AKT (1:500, Cell Signaling Technology), anti-phospho-ERK (1:500, Santa Cruz Biotechnology), anti-H-OXY (1:500, Santa Cruz Biotechnology), anti-i-NOS (1:500, Cell Signaling Technology), anti-HSP60 (1:500, Cell Signaling Technology), anti-GFAP (1:1000, Cell Signaling Technology), anti-NFkB (1:500, Santa Cruz Biotechnology) and anti-β-actin (1:10,000, Sigma-Aldrich). Primary antibodies were detected using the Odyssey^®^ scanner (Li-cor), according to the manufacturer’s instructions, using secondary antibodies (anti-mouse and anti-rabbit) labeled with IR790 and IR680 (1:10,000; Life Technology). Band intensities were analyzed with the Odyssey^®^ CLx Imaging System, and expression was adjusted to β-Actin expression. The protein levels were expressed as intensity relative to control.

### 2.4. Tissue Cholesterol Assay 

To measure cholesterol, 10 mg of brain or liver tissue were homogenized in 100 μL of PBS and processed using the Amplex Red Cholesterol Assay Kit (Life Technology), according to the manufacturer’s instructions. Absorbance was measured by using the iMark™ Microplate Absorbance Reader at 490 nm. The tissue cholesterol concentrations were evaluated using a standard curve, according to the manufacturer’s instructions.

### 2.5. Analysis of Nitrogens Level, Griess Assay

Nitrogens level was analyzed using the Griess fluorometric assay (Promega). 10 mg of brain tissue were homogenized in 100 μL of PBS, and after centrifugation the supernatant was used to test the nitrogen level. The samples were incubated with the Griess reagent in 1:1 ratio at room temperature for 15 min in the dark. Absorbance was measured at 550 nm with a spectrophotometric Microplate reader (WallacVictor2 Multilabel Counter, Perkin Elmer, Rodgau-Jügesheim, Germany). The RNS concentration was evaluated using a standard curve, according to the manufacturer’s instructions.

### 2.6. Quantitative Real-Time PCR

Total RNA was extracted using the RNeasy Lipid Tissue Mini Kit (Qiagen Valencia, CA, USA). Two ng of RNA were used to synthesize the first strand of cDNA, using the RT First-Strand Kit (Qiagen). Synthesized cDNAs were amplified using RT2 SYBR Green/ROX qPCR Mastermix (Qiagen) and the StepOne Real-Time instrument (Applied Biosystem, Foster City, CA, USA). Gene expression validation was performed using home-made sequence primers for human CerS-1 (Forward CGTAAGGACTCGGTGGTCAT, Reverse GCGTAGGAAGAGGCAATGAG), CerS-4 (Forward GATGAAGCCTCTCTGCTGCT, Reverse AGGACACCCACAGGTTTCTG) and 18srRNA (Forward GGACACGGACAGGATTGACA, Reverse ACCCACGGAATCGAGAAAGA). Gene expression was normalized to 18srRNA. On the basis of the *C*_t_ value (threshold cycle: the number of reaction cycles after which fluorescence exceeds the defined threshold) of the examined gene and of the internal control gene, the relative expression level of RNA was calculated, following the 2^−ΔΔ*C*t^ approximation method.

### 2.7. Detection of Oxidative Levels: DCFH-DA Assay

Production of reactive oxygen species (ROS) was evaluated by using 2′,7′-dichlorodiidrofluorescineacetate (DCFH-DA) (Molecular Probes). 5 mg of brain tissue were homogenized in 5 mL of PBS buffer and, after centrifugation, 100 µL of the supernatant were plated on 96-well plates and, after the addition of 1 μL of DCFH-DA, incubated for 5 min. Oxidation levels were evaluated using the GloMax^®^ Discover System (Promega) at 37 °C at an excitation wavelength of 475 nm and an emission wavelength of 555 nm. 

### 2.8. Lipid Peroxidation Assay

10 mg of brain were homogenized in 300 µL of Malondialdehyde (MDA) Lysis Buffer, and the Lipid Peroxidation MDA Assay (Sigma-Aldrich) was used to detect the concentration of lipid peroxidation, according to the manufacturer’s instructions. Absorbance was measured at 532 nm with the iMark™ Microplate Absorbance Reader.

### 2.9. Nile Red Staining

10 mg of brain were homogenized in 1 mL of Nile Red (ThermoFisher Scientific, San Jose, CA, USA) diluted 1:1000 in acetone. The homogenate was incubated at room temperature for 15 min. 2 μL of this solution were spotted on a nitrocellulose membrane, and the fluorescence was visualized using the Typhoon FLA 9500 scanner (excitation/emission 552/636 nm).

### 2.10. Immunofluorescence 

For immunofluorescence, the brain was embedded in paraffin as previously described [14] and coronally sectioned (5 μm) using a microtome. Brain sections, including the cerebral cortex, corpus callosum, hippocampus, thalamus and hypothalamus, were mounted on slides and deparaffinized in xylene solution. Then, the slides were hydrated in a series of graded ethanol (96%, 85%, 70%, 50%) for 5 min each. After washing in water and PBS, the slides were incubated with 3% BSA/PBS for 1 h. Next, the sections were incubated with anti-phospho-ERK (1:50, Santa Cruz Biotechnology) and anti-GFAP (1:50, Cell Signaling Technology), respectively, at 4 °C overnight. After washing in PBS, the samples were incubated with anti-rabbit Cy3-conjugate secondary antibody (1:500; SIGMA). After washing in PBS, the slides were mounted with cover slips, and the images were visualized using a Leica DM5000 upright microscope (Leica Microsystems, Heidelberg, Germany) at 20× magnification.

### 2.11. Immunohistochemistry 

Sections (5 μm thick) of paraffined-embedded brains were hydrated in a sequence of graded ethanol (from 96% to 50%) for 5 min each, washed in water and then PBS. The slides were incubated with 3% BSA for 1 h and subsequently, with anti-CD4+ (1:40) (Dako) at 4 °C overnight. After washing, the LSAB2 Dako Kit (Dako) and Fuchsin Substrate-Chromogen System (Dako) were used for brown staining. The slides were mounted with cover slips, and images were visualized using a Leica DM5000 upright microscope (Leica Microsystems) at a magnification of 20×.

### 2.12. Nuclear Staining

Brain paraffin sections (5 μm thick) were hydrated in a series of graded ethanol (96%, 85%, 70%, 50%) for 5 min each, washed in water and PBS, incubated with Hoechst 33258 (5 μg/mL) for 20 min. Images were visualized using a Leica DM5000 upright microscope (Leica Microsystems) at 20× magnification, Ex 460 nm/Em 490 nm at the excitation wavelength of 475 nm and emission wavelength of 555 nm.

### 2.13. Tunel Assay

Terminal deoxynucleotidyl Transferase Biotin-dUTP Nick End Labeling (TUNEL)-positive apoptotic nuclei were detected in paraffinized brain sections using an in-situ cell death detection kit (Promega), according to the manufacturer’s instructions. Briefly, paraffinized brain sections, including the cerebral cortex, corpus callosum, hippocampus, thalamus and hypothalamus, were mounted on slides and deparaffinized at 60 °C for 1 h, followed by xylene solution for 5 min. Then, the slides were hydrated in a series of graded ethanol (96%, 85%, 70%, 50%) for 5 min each and washed in PBS and incubated with a TUNEL reaction mixture (enzyme and nucleotide) in a humified atmosphere at 37 °C for 1 h. Staining was obtained by using a peroxidase substrate, hydrogen peroxide, and the stable chromogen diaminobenzidine (DAB). After this incubation, the samples were rinsed three times with PBS and analyzed under a Zeiss Axio Scope microscope (ZEISS). The number of apoptotic cells was counted in randomly selected fields to calculate the ratio of apoptotic cells to brain area.

### 2.14. ELISA Assay

10 mg of brain tissue were homogenized in 10 mL of buffer (supplied in the ELISA kit), after centrifugation at 14,000 RPM for 30 min at 4 °C. 100 µL of the supernatant were used for determining interleukin-1β (Thermo Fisher Scientific, Rockford, IL, USA) and interleukin-6 (Cloud-Clone Corp., Houston, TX, USA), according to the manufacturer’s instructions. Briefly, 100 μL of the samples were incubated in duplicate for 1 h at 37 °C in the supplied multiwell, followed by the addition of prepared “detection reagent A” (100 µL) and incubation for 1 h at 37 °C. Then, the solution was eliminated and the multiwell washed 3 times with washing buffer. 100 µL of prepared “detection reagent B” were added and incubated for 30 min at 37 °C. After washing 3 times in washing buffer, 90 μL of “substrate solution” were added and incubated for 15 min at 37 °C. The reaction was stopped with 50 μL of “stop solution” and read at 450 nm immediately with the iMark™ Microplate Absorbance Reader. As a reference for quantification, a standard curve was made.

### 2.15. Statistical Analyses

The results are presented as mean ± SD. A one-way ANOVA was performed, followed by Dunnett’s post hoc test for analysis of significance. Results with a *p*-value < 0.05 were considered statistically significant, * *p* < 0.05, versus STD group. # *p* < 0.05 versus HFD group.

## 3. Results

### 3.1. NDS Prevents HFD-Induced Dysmetabolism

**Figure 1:**
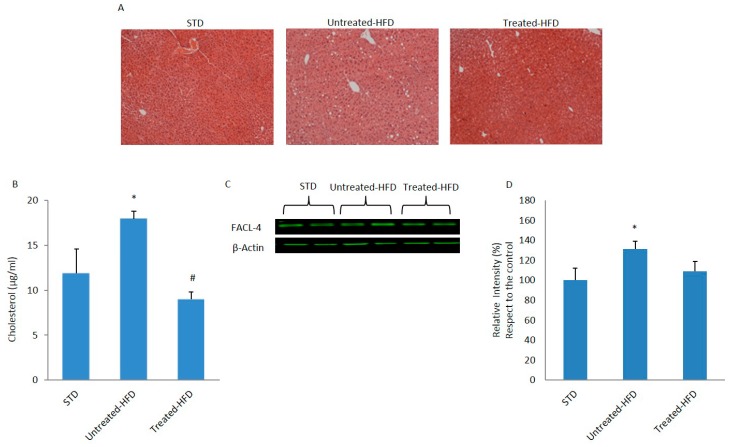
Effects of NDS on the liver. (**A**) Liver sections of control (STD), HFD (untreated-HFD) and NDS-treated (treated-HFD) mice; (**B**) Intrahepatic cholesterol concentration; (**C**) Western blot of proteins extracted from livers of STD, untreated-HFD and NDS treated-HFD mice and incubated with anti-fatty acid-CoA ligase-4 (FACL-4) and anti-β-Actin (loading control); (**D**) Quantification of immunoreactivity was performed using densitometric analysis. Data are the mean values ± SEM (*n* = 8/group). * *p* ≤ 0.05 vs. STD; # *p* ≤ 0.05 vs. untreated-HFD.

As shown in Table 2, and in accordance with previous data [29], NDS prevented increases in body weight and circulating lipids, observed by comparing untreated HFD mice with STD animals. In the NDS treated-HFD group, all the parameters were similar to the STD mice. Moreover, histological analysis revealed the presence of micro- and macro-vesicular steatosis in the livers of untreated HFD mice. A slight accumulation of fat was observed in the NDS treated-HFD group, confirming the hepatic protective effects previously described [29] (Figure 1A). In addition, an increase in cholesterol concentration was observed only in the livers of untreated HFD mice (Figure 1B). The expression of fatty acid-CoA ligase-4 (FACL-4), an enzyme involved in lipid biosynthesis and fatty acid degradation, was analyzed. In fact, the enzyme converts free fatty acids into fatty acyl-CoA esters, which are key intermediates in the synthesis of complex lipids. FACL-4 expression was significantly higher in the livers of untreated HFD mice than in STD or NDS treated-HFD mice (Figure 1C,D).

### 3.2. Brain Lipid Accumulation is Prevented by NDS

**Figure 2:**
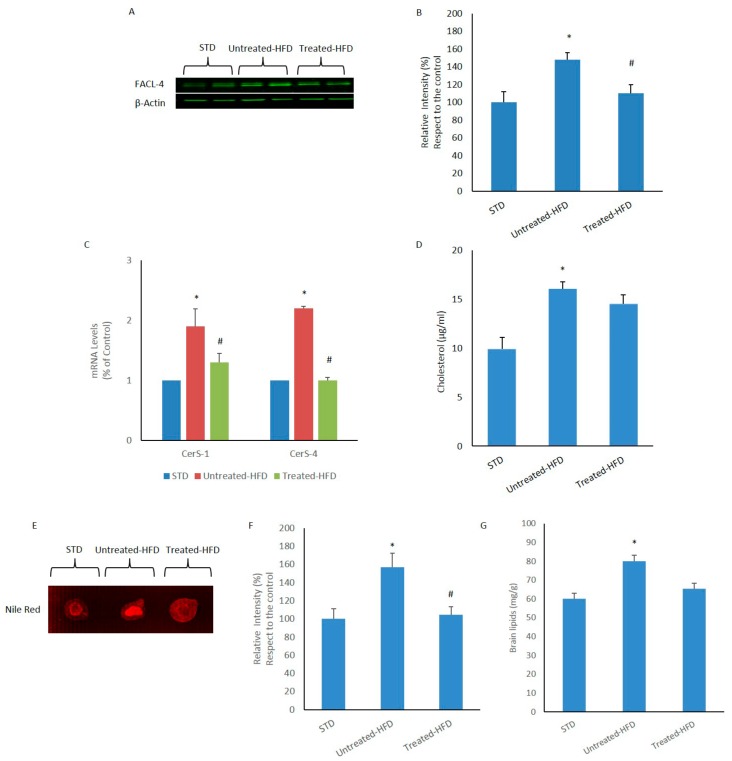
Lipid accumulation in the brain. (**A**) Western blot of proteins extracted from brains of STD, untreated-HFD and NDS treated-HFD mice and incubated with anti-FACL-4 and anti-β-Actin (loading control); (**B**) Quantification of immunoreactivity was performed using densitometric analysis; (**C**) CerS-1 and CerS-4 transcript levels determined by quantitative Real-Time PCR; (**D**) Cholesterol concentration in the brain tissue; (**E**) Nile Red staining of brain homogenates; (**F**) Quantification of E fluorescence intensity; (**G**) Total brain lipid levels. Data are the mean values ± SD (*n* = 8/group). * *p* ≤ 0.05 vs. STD. # *p* ≤ 0.05 vs. untreated-HFD.

The expression of FACL-4 was also analyzed in the brain. Western blotting experiments showed increased expression in brains of untreated-HFD mice, in comparison with STD control. In contrast, the expression of FACL-4 in NDS treated-HFD mice was similar to the control (Figure 2A,B). We also measured the expression level of the CerS-1 and CerS-4 genes involved in the *de novo* ceramide synthesis. qRT-PCR analysis revealed higher levels of CerS-1 and CerS-4 mRNA transcripts in the brains of untreated HFD-fed mice compared to STD and NDS treated-HFD mice (Figure 2C). Moreover, increased cholesterol concentration in the brains of untreated-HFD mice was found, whereas the cholesterol level in the cerebral tissue of NDS-treated animals was similar to STD mice (Figure 2D). Finally, lipid levels were measured by staining brain homogenates with Nile Red, a hydrophobic fluorescent probe which displays fluorescence in the presence of lipids. Fluorescence increased signals was only evident in untreated-HFD, being lipid levels similar to STD control. All these data suggest that an alteration in lipid metabolism can be inhibited by NDS treatment. 

### 3.3. NDS Decreases Insulin Signaling Alteration

**Figure 3:**
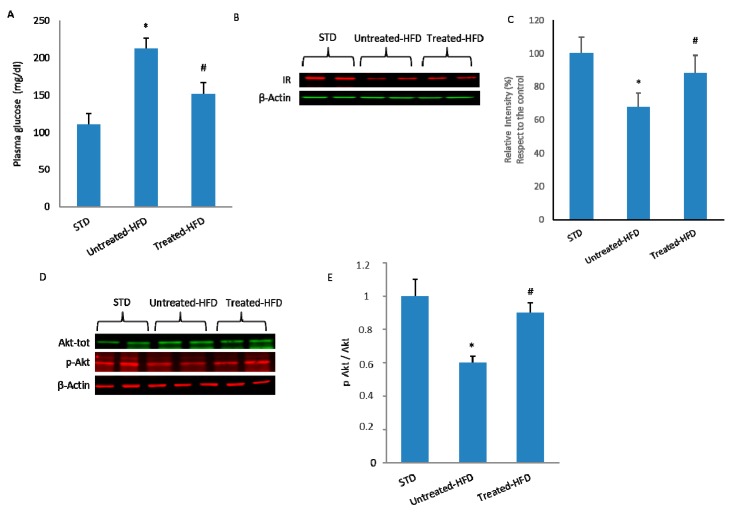
NDS decreases the HFD-induced insulin signaling alterations in the brain. (**A**) Fasting plasma glucose concentration in STD, untreated-HFD and treated-HFD mice; (**B**) Western blot of proteins extracted from brains of STD, untreated-HFD and NDS treated-HFD mice and incubated with anti-Insulin Receptor (IR) and anti-β-Actin (loading control); (**C**) Quantification of immunoreactivity was performed using densitometric analysis; (**D**) Western blot of proteins extracted from brains of STD, untreated-HFD and NDS treated-HFD mice and incubated with anti-Akt, anti-phospho-Akt (p-Akt) and anti-β-Actin (loading control); (**E**) Quantification of Akt/p-Akt ratio. Data are the mean values ± SD (*n* = 8/group). * *p* ≤ 0.05 vs. STD. # *p* ≤ 0.05 vs. untreated-HFD.

The untreated-HFD mice showed a significant increase in fasting plasma glucose concentration in comparison with STD animals. In NDS treated-HFD mice, fasting glucose concentration was significantly lower than untreated-HFD animals (Figure 3A). Since insulin resistance is characterized by a reduced number of receptors [12] and the downregulation of insulin signaling [13], IR expression and Akt activation levels were measured and compared in the brains of different groups. HFD mice showed significantly lower IR expression than STD mice; in contrast, in the NDS treated-HFD group, the brain IR expression level was comparable to that of STD-fed mice (Figure 3B,C). Furthermore, to investigate whether insulin signaling was affected, the expression of total and phosphorylated forms of Akt were analyzed. The p-AKT/AKT ratio showed a decrease in the brains of untreated-HFD mice. In contrast, in the brains of NDS treated-HFD mice, the phospho-Akt/Akt ratio was less reduced in comparison with untreated-HFD animals (Figure 3D,E), suggesting that brain insulin resistance is present only in untreated obese mice. 

### 3.4. NDS Prevents Oxidative Stress and Lipid Peroxidation

**Figure 4:**
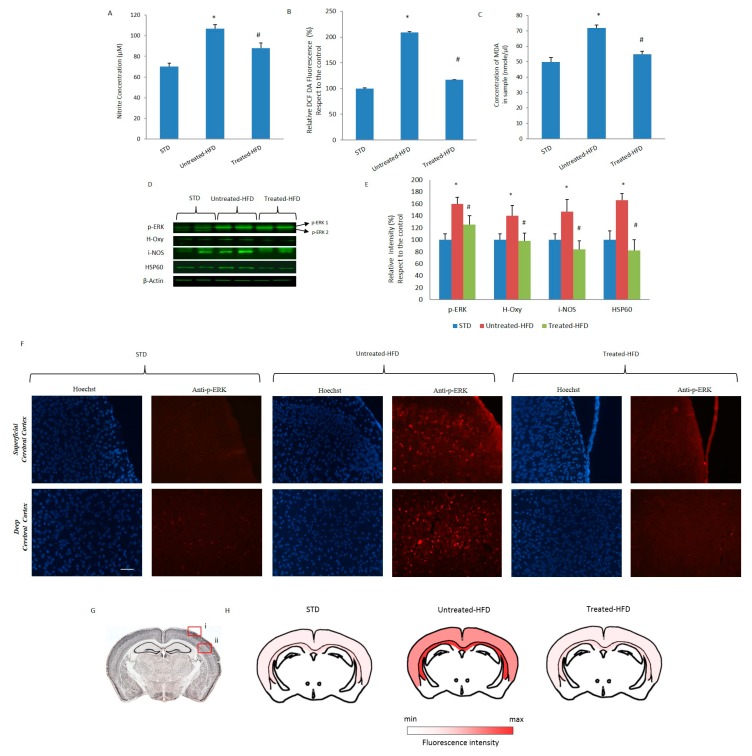
Nitric and oxidative stress and lipid peroxidation in the brains of HFD-mice were prevented by NDS treatment. (**A**) Nitrite concentration in STD, untreated-HFD and treated-HFD brains; (**B**) Levels of ROS in STD, untreated-HFD and treated-HFD brains (**C**) Lipid peroxidation levels in STD, untreated-HFD and treated-HFD brains; (**D**) Western blot of proteins extracted from brains of STD, untreated-HFD and treated-HFD mice and incubated with anti-p-ERK, anti-H-Oxy, anti-i-NOS, anti-HSP60 and anti-β-Actin (loading control); (**E**) Quantification of immunoreactivity was performed using densitometric analysis; (**F**) Immunofluorescence of superficial and deep cerebral cortex sections of STD, untreated-HFD and treated-HFD mice incubated with anti-phospho-ERK; (**G**) Schematic representation of superficial (i) and deep (ii) cerebral cortex positive areas; (**H**) Brain map indicating the levels of positive p-ERK staining. Representative images from 3 animals per group are shown. Bar 20 μm. Data are the mean values ± SD (*n* = 8/group). * *p* ≤ 0.05 vs. STD. # *p* ≤ 0.05 vs. untreated-HFD.

In dysmetabolic conditions, hyperglycemia, dyslipidemia and insulin resistance are often associated with oxidative stress and lipid peroxidation. By using specific assays, we observed that oxidative conditions (nitrogens level, ROS and lipid peroxidation) in the brains of untreated-HFD mice were increased with respect to the STD group (Figure 4). On the contrary, in the brains of NDS-treated HFD mice, the levels of nitrogens, ROS and lipid peroxidation were lower than in untreated-HFD mice (Figure 4A–C), suggesting an antioxidant effect of NDS. Moreover, we analyzed the expression of other stress biomarkers. We found increased expression of phospho-ERK (p-ERK), heme oxygenase (H-Oxy), induced-NOS (i-NOS) and HSP60 only in the brains of untreated-HFD mice compared to the STD group. In fact, NDS treated-HFD mice showed the expression of these biomarkers at similar levels to STD animals (Figure 4E,D). The absence of increased expression of phospho-ERK in NDS treated-HFD mice was confirmed by immunofluorescence experiments. As shown in Figure 4F, p-ERK immunoreactivity was less prominent in superficial and deep cerebral cortex sections of NDS treated-HFD mice. The levels of p-ERK in each brain region were represented (Figure 4G) as a heat map generated on the basis of the data obtained from the microscopy fluorescence. 

### 3.5. NDS Decreases the Hdf-Induced Brain Inflammation Profile

**Figure 5:**
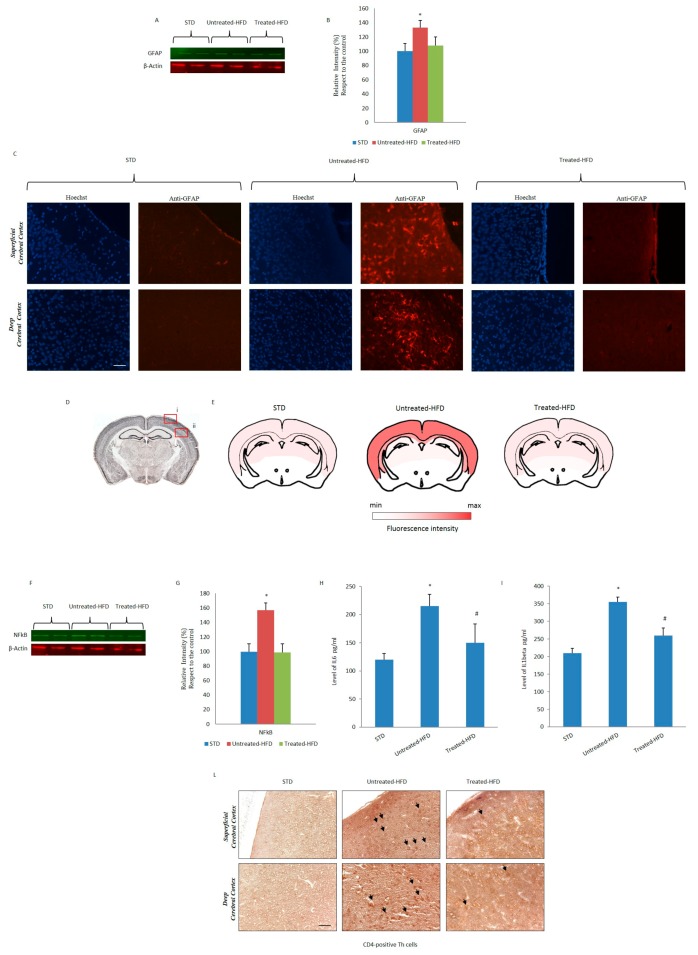
NDS prevents HFD-induced inflammation and immunological response in the brain. (**A**) Western blot of proteins extracted from brains of STD, untreated-HFD and treated-HFD mice and incubated with anti-GFAP and anti-β-Actin (loading control); (**B**) Quantification of immunoreactivity was performed using densitometric analysis; (**C**) Immunofluorescence of superficial and deep cerebral cortex sections incubated with anti-GFAP; (**D**) Schematic representation of superficial (i) and deep (ii) cerebral cortex positive areas; (**E**) Brain map indicating the levels of positive staining of GFAP; (**F**) Western blot of proteins extracted from brains of STD, untreated-HFD and treated-HFD mice and incubated with anti-NFkB and anti-β-Actin (loading control); (**G**) Quantification of immunoreactivity was performed using densitometric analysis; (**H**,**I**) Levels of IL-6 and IL-1β in brains of STD, untreated-HFD and treated-HFD mice, quantified by ELISA assay. (**L**) Brain sections from STD, untreated-HFD and treated-HFD mice incubated with anti-CD4. Representative images from 3 animals per group are shown. Bar 20 μm. Data are the mean values ± SD (*n* = 8/group). * *p* ≤ 0.05 vs. STD. # *p* ≤ 0.05 vs. untreated-HFD.

Brain inflammation was investigated by analyzing several biomarkers. The expression of GFAP, a gliosis specific marker detected by Western blot analysis, was increased in untreated-HFD mice compared to STD and NDS treated-HFD mice (Figure 5A,B). This observation was confirmed by immunofluorescence experiments, with GFAP positivity being prominent only in cerebral cortex sections from untreated-HFD mice (Figure 5C,D). Increased expression of NF-kB, IL-6 and IL-1β were only detected in untreated-HFD mice, with the values in NDS-treated HFD animals being similar to STD (Figure 5F–H). Moreover, infiltration of inflammatory immune cells into the cortical sections was observed only in HFD-fed mice. CD4^+^ positive cells were revealed by immunohistochemistry (Figure 5L) suggesting anti-inflammatory properties of NDS. 

### 3.6. NDS Counteracts Hfd-Induced Neurodegeneration

**Figure 6:**
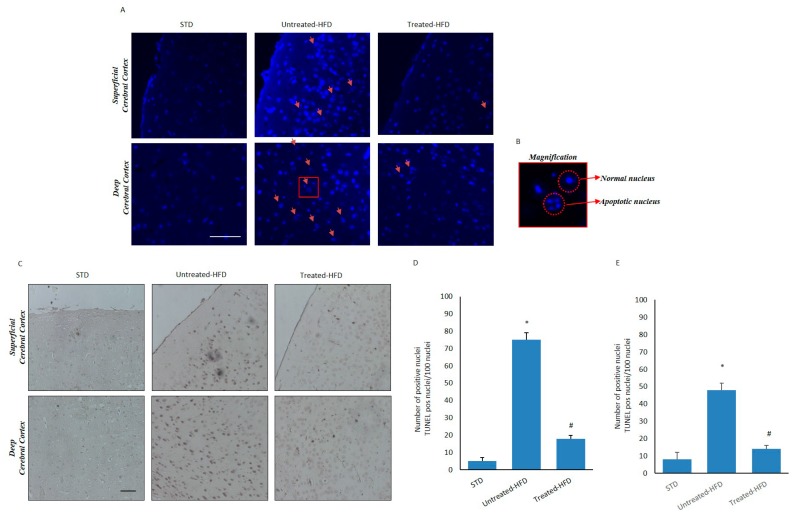
NDS inhibits neurodegeneration induced by HFD. (**A**) Brain sections from STD, untreated-HFD and treated-HFD mice incubated with Hoechst 33342. Fragmented apoptotic nuclei are indicated by the arrows; (**B**) The outlined area is enlarged in the squares; (**C**) TUNEL assay on superficial and deep cortical regions from STD, untreated-HFD and treated-HFD mice; (**D**) Number of apoptotic nuclei in the superficial cerebral cortex positive area; (**E**) Number of apoptotic nuclei in the deep cerebral cortex positive area. Representative images from 3 animals per group are shown. Bar 20 μm. Data are the mean values ± SD (*n* = 8/group). * *p* ≤ 0.05 vs. STD. # *p* ≤ 0.05 vs. untreated-HFD.

Because cellular stress and inflammation can trigger neurodegeneration [30], we analyzed the number of apoptotic cells by using both Hoechst staining (Figure 6A,B) and TUNEL enzymatic assay (Figure 6C) in superficial and deep cortical sections. Fragmented nuclei were more present in both analyzed areas of untreated-HFD mice in comparison with NDS-treated HFD mice. In addition, TUNEL-positive cell numbers were significantly higher in both cortical sections of untreated-HFD mice compared to STD- and treated-HFD animals (Figure 6D,E), suggesting a decrease of apoptotic cellular death in treated animals. 

## 4. Discussion

In the present study, we have demonstrated, for the first time, that a natural dietary supplement, well-known as a hepatoprotector, is also able to exert neuroprotective effects on mice with diet-induced obesity. The NDS containing curcumin, silymarin, guggul, chlorogenic acid and inulin achieves its function by reducing brain fat accumulation, oxidative stress and inflammation, and by improving brain insulin resistance. 

While aging is clearly the strongest risk factor for neurodegenerative diseases such as AD, emerging data suggest that dysmetabolic conditions associated with obesity, such as hyperglycemia, insulin resistance, dyslipidemia, oxidative stress and inflammatory state, can act as co-factors in neurodegenerative pathogenesis [14,31,32]. We used an animal model (HFD mice) which rapidly increases body weight, develops hyperglycemia [33], hepatic steatosis [34], neuroinflammation and neurodegeneration [14,35,36], and is well-suited for testing compounds useful in MeS therapy and other related pathologies. 

Considering the lack of a single suitable remedy for treating both metabolic dysfunctions and associated neurodegenerative diseases, current therapeutic approaches are focused on natural solutions. In particular, an increasing number of studies suggest that the dietary intake of vegetables and fruits can prevent or delay the onset of neurodegenerative diseases [36] due to the presence of flavonoids and phenolic compounds with antioxidant properties [37,38].

The natural dietary supplement used for our experiments is known to exert beneficial effects on different components of MetS, such as insulin resistance, glucidic metabolism [39], dyslipidemia [40] and neurodegeneration. In particular, curcumin has been reported to have anti-neuroinflammatory and neuroprotective effects on AD pathogenesis in rats, through the activation of PPARγ [41]; silymarin is able to inhibit β-amyloid (Aβ) protein self-assembly, showing a potential protective effect in AD pathogenesis [42], chlorogenic acid protects against rat cortical neuron degeneration associated with oxidative stress [43], and inulin increases fecal concentrations of tyramine and cytotoxic tryptamine [44]. Our recent work has demonstrated that NDS is able to prevent dyslipidemia, liver steatosis and atherosclerosis in obese HFD mice by modulating gene expression in the liver [29]. 

In the present study, the beneficial effects of NDS against dysmetabolism were extended to the brain. After 16 weeks, the ability of NDS to prevent HFD-induced steatosis was ascertained by the reduction of plasma triglycerides, total cholesterol and LDL, and increasing HDL concentration. Moreover, in NDS treated mice, lower hepatic cholesterol concentration and FACL-4 expression were observed, confirming the ability of NDS to prevent liver fat accumulation. Since altered lipid homeostasis can lead to neuronal injury [8,45], the expression of enzymes involved in lipid and ceramide biosynthesis and total lipids were evaluated in brains of the different mouse groups. In our experiments, we observed the increased expression of FACL-4, CerS-1 and CerS-4 and a higher cholesterol concentration in the brains of untreated HFD mice, confirming an increase of fat content.

In contrast, the antilipidemic benefits of NDS was extended to the brains of obese animals. Although the relative proportion of lipids which were of circulating origin or locally produced in the brain remains unclear, our results suggest that increased *de novo* lipid biosynthesis by the brain was prevented by NDS. An upregulation of CerS-1, CerS-4 and SMPD pro-ceramide gene expression was, indeed, detected in the brain tissue of HFD-fed mice, in agreement with the increase of ROS levels and neurodegenerative neurons [46]. In contrast, in animals receiving dietary supplementation, we found that the *de novo* expression of the pro-ceramides was similar at a basal level, consistent with the absence of metabolic dysfunction.

As occurs in peripheral tissues, the accumulation of lipid species, such as free fatty acids, cholesterol, and ceramides, could contribute to inducing insulin resistance via Akt inhibition in the brain [18,47,48]. Accumulation of cholesterol was found in AD amyloid [49], and other neurodegenerative disorders are associated with abnormal lipid metabolism and deposition [50]. Similarly, we found that alterations in lipid metabolism correspond to an increase of apoptotic nuclei in brain sections of HFD mice and that NDS co-administration prevents neurodegenerative events. Thus, NDS protects the brain from dysfunction caused by alterations in lipid metabolism due to HFD. Lipid and cholesterol lowering can be attributed to the presence of guggul, which inhibits the biosynthesis of cholesterol in the liver and, probably, in the brain as well [51].

With this in mind, we investigated the effects of chronic NDS treatment on central insulin resistance. Interestingly, our results demonstrated that NDS is able to prevent the increase of fasting glycemia observed in obese mice. This NDS antihyperglycemic effect fits well with the antidiabetic properties of silymarin, curcumin, chlorogenic acid, and inulin [52,53,54]. Moreover, in the brains of obese mice, IR and p-Akt expression were decreased in comparison with STD mice, suggesting the presence of insulin resistance. However, NDS treated HFD mice showed significantly increased expression of IR and p-Akt compared to the untreated obese group, suggesting a protective effect of NDS against the development of cerebral insulin resistance. 

Dyslipidemia, hyperglycemia, and insulin resistance are often associated with other dysfunctions, such as oxidative stress and lipid peroxidation, that in turn could be involved in neurodegenerative processes [55]. For this purpose, the potential antioxidant effect of the NDS was analyzed and compared in the brains of different animals. All the analyzed oxidative parameters like NO, ROS, MDA and stress biomarkers, such as phospho-ERK, Heme Oxygenase, i-Nos and HSP60, were affected by diet and maintained approximately at a basal level after NDS co-administration. These results could be due to the antioxidant activities described for the components of the natural extracts present in the NDS [56,57]. Since the vulnerability of the central nervous system to reactive oxygen species is well established, it can easily be understood how important it is that a dietary supplement be able to maintain oxidative stress at homeostatic levels.

A beneficial effect of NDS was also observed in preventing neuroinflammation, a deleterious pathological condition involved in neurodegenerative diseases. According to previous studies [58,59], HFD intake and obesity induce glia activation and central neural inflammation, as demonstrated by an increase of the gliosis marker GFAP as well as pro-inflammatory mediators (NF-kB, IL-6 and IL-1β) and the infiltration of immune cells. All these markers were reduced in the brains of the NDS-treated obese group. Considering that NF-kB acts as a transcription factor for different pro-inflammatory cytokines, including IL-6 and IL-1β [60], we could speculate that the anti-inflammatory effect of NDS could be due to reduced NF-kB expression. Furthermore, we cannot exclude that this effect could be attributed to curcumin, which is known to exert a potent protective role against neuro-inflammation in the brains of obese rats [41]. In agreement with the protective efficacy of NDS against brain cholesterol and ceramide accumulation, impaired central insulin signaling, increased oxidative stress and inflammatory status, our results demonstrate that NDS is able to prevent neuronal apoptosis. In fact, the high number of neurons with fragmented DNA, a marker of cell death, present in the brains of the HFD-untreated animals, was not detected in the brains of NDS-treated-HFD animals. 

## 5. Conclusions

The results of the present study provide evidence for a new potential therapeutic use of the NDS aimed at preventing central dysmetabolic conditions leading to neurodegeneration, such as cholesterol and ceramide accumulation, insulin resistance, oxidative stress and neuroinflammation. We maintain that the assimilation of the ingredients present in the NDS, probably acting synergistically on different metabolic pathways, can have beneficial effects in both the liver and the brain.

## Figures and Tables

**Table 1:** Composition and energy densities of standard laboratory diet (STD) and high-fat diet (HFD).

Ingredient (g/kg)	STD	HFD
Acid Casein 741	200	265.00
l-Cystine	2.8	4
Maltodextrine—0032	33.2	160
Sucrose	300	90
Cellulose (Arbocel)	50	65.5
Soybean oil	25	30
Lard	19	220
Vitamin mix AIN-93-VX-PF2439	10	13
Mineral mix AIN-93G-MX-PF2348	45	48
Choline bitartrate	1.9	3
Calcium Phosphate dibasic	13	3.4
**Total Energy**		
Kcal/g	3.5	6
Protein, %	18.5	23
Carbohydrate, %	60	38
Fat, %	3	34

**Table 2:** Effects of natural dietary supplement (NDS) on high fat diet (HFD)-induced dysmetabolism.

	STD	Untreated-HFD	NDS Treated-HFD	*p*-Value
Body Weight (g)	25.1 ± 1.1	33.1 ± 0.66 *	27.1 ± 0.7 ^#^	* *p* < 0.05; # *p* < 0.05
TG (mg/dL)	49 ± 4.2	68.7 ± 3.2 *	55 ± 2.9 ^#^	* *p* < 0.05; # *p* < 0.05
Cholesterol (mg/dL)	85.8 ± 4.8	112.6 ± 5.2 *	90 ± 7.1 ^#^	* *p* < 0.05; # *p* < 0.05
LDL	37 ± 5.2	62.6 ± 5.5 *	43 ± 4.3 ^#^	* *p* < 0.05; # *p* < 0.05
HDL	52.8 ± 4.2	36 ± 3.8 *	49.5 ± 2.9 ^#^	* *p* < 0.05; # *p* < 0.05

Data are the mean values ± SD (*n* = 8/group). * *p* ≤ 0.05 vs. STD; # *p* ≤ 0.05 vs. untreated-HFD.

## Supplementary Materials

The following are available online at http://www.mdpi.com/2072-6643/10/9/1130/s1.
