# Supplementary material for: A Natural Dietary Supplement with a Combination of Nutrients Prevents Neurodegeneration Induced by a High Fat Diet in Mice
Source: Nutrients. 2018 Aug 21;10(9):1130. doi: 10.3390/nu10091130 (PMC6165339; doi:10.3390/nu10091130)
Supplement: Supplementary file 1 [file nutrients-10-01130-s001.pdf]

Supplemental Table 1. Composition of mineral mix in STD and HFD

PMIX AIN-93G-MX	
**Ingredient (mg/kg)**	**4800 g/100 kg**
Calcium carbonate	6854.40
Potassium phosphate monobasic	2700.10
Potassium citrate tribasic monohydrate	1223.08
Sodium chloride	1395.94
Magnesium oxide	703.34
Potassium sulfate	1004.32
Chromium K sulfate	1.37
Cupric carbonate	8.26
Sodium fluoride	1.38
Potassium iodate	0,28
Ferric citrate	55.27
Manganese carbonate	14.45
Ammonium molybdate	0.21
Basic nickel carbonate	0.15
Lithium chloride	0.14
Boric acid	0.68
Ammonium metavanadate	0.14
Sodium metasilicate	6.88
Zinc carbonate	45.94
Sodium selenite	0.21

Abbreviations are: STD, standard diet; HFD, High Fat Diet

Supplemental Table 2. Composition of vitamin mix in STD and HFD

PMIX AIN-93G-MX		
**Ingredient**	**2100 g/100 kg**	
Vit. K_1 phylloquinone 97-103%	1.58	**mg/Kg**
Nicotinic acid 99.5-100.5%	63.0	**mg/Kg**
Calcium pantothenate 98-101%	33.6	**mg/Kg**
Vit. A palmitate 250	8400	**I.U./Kg**
Biotin 97.5-100.5%	0.42	**mg/Kg**
Piridoxine 99-101%	14.70	**mg/Kg**
Riboflavin 97-103%	12.60	**mg/Kg**
Thiamine 98.5-101%	12.60	**mg/Kg**
Vit. D3 Cholecalciferol, 500	2100	**I.U./Kg**
Cyanocobalamin > 0.1%	0.053	**mg/Kg**
Folic Acid 96-102%	4.16	**mg/Kg**
α-tocopheryl acetate, 500 IU/g	157.50	**mg/Kg**
